# Editorial Note: Long-term consequences of benzodiazepine-induced neurological dysfunction: A survey

**DOI:** 10.1371/journal.pone.0352305

**Published:** 2026-06-24

**Authors:** The PLOS One Editors

The *PLOS One* Editors issue this Editorial Note because this article [1] was identified as one of a series of submissions for which we have concerns about aspects of the peer review process. Readers are advised to be aware of these peer review concerns when considering the article [1]. The article’s contents and findings were not assessed or questioned by the Editors as part of the investigation.

The Editors do not have concerns regarding the authors’ contributions to peer review.
